# Photosynthetic limitations in two Antarctic vascular plants: importance of leaf anatomical traits and Rubisco kinetic parameters

**DOI:** 10.1093/jxb/erx148

**Published:** 2017-05-10

**Authors:** Patricia L Sáez, León A Bravo, Lohengrin A Cavieres, Valentina Vallejos, Carolina Sanhueza, Marcel Font-Carrascosa, Eustaquio Gil-Pelegrín, José Javier Peguero-Pina, Jeroni Galmés

**Affiliations:** 1 Laboratorio Cultivo de Tejidos Vegetales, Centro de Biotecnología, Departamento de Silvicultura, Facultad de Ciencias Forestales, Universidad de Concepción, Concepción, Chile; 2 Laboratorio de Fisiología y Biología Molecular Vegetal, Instituto de Agroindustria, Departamento de Ciencias Agronómicas y Recursos Naturales, Facultad de Ciencias Agropecuarias y Forestales, Center of Plant, Soil Interaction and Natural Resources Biotechnology, Scientific and Technological Bioresource Nucleus, Universidad de La Frontera, Temuco, Chile; 3 Laboratorio de ECOBIOSIS, Departamento de Botánica, Facultad de Ciencias Naturales y Oceanográficas, Universidad de Concepción, Barrio Universitario s/n, Concepción, Chile; 4 Research Group on Plant Biology under Mediterranean Conditions, Universitat de les Illes Balears-INAGEA, Balearic Islands, Spain; 5 Unidad de Recursos Forestales, Centro de Investigación y Tecnología Agroalimentaria, Gobierno de Aragón, Zaragoza, Spain

**Keywords:** Antarctic plants, leaf traits, mesophyll conductance, photosynthesis, Rubisco, temperature

## Abstract

Particular physiological traits allow the vascular plants *Deschampsia antarctica* Desv. and *Colobanthus quitensis* (Kunth) Bartl. to inhabit Antarctica. The photosynthetic performance of these species was evaluated *in situ*, focusing on diffusive and biochemical constraints to CO_2_ assimilation. Leaf gas exchange, Chl *a* fluorescence, leaf ultrastructure, and Rubisco catalytic properties were examined in plants growing on King George and Lagotellerie islands. In spite of the species- and population-specific effects of the measurement temperature on the main photosynthetic parameters, CO_2_ assimilation was highly limited by CO_2_ diffusion. In particular, the mesophyll conductance (*g*_m_)—estimated from both gas exchange and leaf chlorophyll fluorescence and modeled from leaf anatomy—was remarkably low, restricting CO_2_ diffusion and imposing the strongest constraint to CO_2_ acquisition. Rubisco presented a high specificity for CO_2_ as determined *in vitro*, suggesting a tight co-ordination between CO_2_ diffusion and leaf biochemistry that may be critical ultimately to optimize carbon balance in these species. Interestingly, both anatomical and biochemical traits resembled those described in plants from arid environments, providing a new insight into plant functional acclimation to extreme conditions. Understanding what actually limits photosynthesis in these species is important to anticipate their responses to the ongoing and predicted rapid warming in the Antarctic Peninsula.

## Teaser

Antarctic vascular plants show restrictions in mesophyll CO_2_ conductance due to morphological leaf adaptations to deal with the harsh climate. Rubisco kinetic parameters, however, compensate by optimizing photosynthesis and carbon balance.

## Introduction

Antarctica is the coldest, driest, and windiest continent on Earth, with mean summer air temperatures <0 °C in continental Antarctica and between 0 and 2 °C in Maritime Antarctica (Convey, 1996). The study of how organisms behave in this hostile habitat is of particular interest to unravel functional adaptations to extreme conditions (see Alberdi *et al.*, 2002; Cavieres *et al.*, 2016).

*Deschampsia antarctica* Desv. (Poaceae) and *Colobanthus quitensis* (Kunth) Bartl. (Caryophyllaceae) are the only two vascular plants naturally colonizing these harsh conditions, being distributed along the west coast of the Antarctic Peninsula and its associated islands (Maritime Antarctica) down to Lazarev Bay on the north-west coast of Alexander Island (69°22.0'S, 71°50.7'W) (Convey *et al.*, 2011). Although several differences have already been described between these two species in dealing with the Antarctic conditions (Pérez-Torres *et al.*, 2007; Cavieres *et al.*, 2016), the performance of both Antarctic vascular species relies on a robust CO_2_ assimilation machinery, including a high activation state of Rubisco and stromal fructose-1,6-biphosphatase (Pérez-Torres *et al.*, 2006). *Deschampsia antarctica* is more abundant and widely distributed along the Antarctic Peninsula than *C. quitensis* (Smith, 2003), colonizing different habitats ranging from mineral to organic soils, and from dry to waterlogged areas. In contrast, *C. quitensis* seems to be less tolerant to extreme conditions, preferring sparsely vegetated and sheltered sites with moist, but well-drained mineral soils (Smith, 2003). Although both species exhibit CO_2_ assimilation rates similar to those of other herbaceous species from low-temperature environments such as alpine or arctic habitats (Chapin and Shaver, 1985; Körner, 2003), *D. antarctica* has a higher photosynthetic capacity than *C. quitensis* (Montiel *et al.*, 1999; Xiong *et al.*, 1999). Under laboratory conditions, *D. antarctica* and *C. quitensis* are able to maintain ~30% of their maximum photosynthetic rates at 0 °C, but both species display maximal CO_2_ assimilation rates at 13 °C and 19 °C, respectively (Edwards and Smith, 1988). These findings led Xiong *et al.* (1999) to suggest that current and future warming would be beneficial for the carbon assimilation of these two Antarctic plants.

Although some information has been accrued on the responses of the Antarctic plants to the combination of cold and high light, and to the effect of temperature on their photosynthetic performance, most of these data come from laboratory-grown plants. Field assessments on the mechanisms involved in the photosynthetic regulation, on the anatomical and biochemical limitations to photosynthesis, and whether these limitations change among populations exposed to different environmental conditions are fundamental to understand the functional adaptations that these species have evolved to withstand the Antarctic conditions. This knowledge will equally provide powerful insights to anticipate how these species might respond to climate change.

Leaf mesophyll conductance of CO_2_ (*g*_m_) and Rubisco kinetic parameters are two key players in carbon assimilation needed for a proper understanding of the photosynthetic performance under field conditions. The kinetic traits describing Rubisco functioning differ among species (e.g. Delgado *et al.*, 1995; Kubien and Sage, 2008; Hermida-Carrera *et al.*, 2016; Orr *et al.*, 2016), with habitat-dependent variations in some parameters, mostly related to contrasting thermal environment, CO_2_ concentration, and water availability (Galmés *et al.*, 2005, 2014*a*, 2016). Regarding *g*_m_, several studies have quantified the importance of leaf anatomical traits in determining *g*_m_ and, consequently, the photosynthetic capacity of different plant species (Terashima *et al.*, 2001; Tomás *et al.*, 2013; Flexas *et al.*, 2016), or within the same species but growing under different environmental conditions (Peguero-Pina *et al.*, 2015*a*). Contrasting environmental conditions can induce changes in several leaf traits that affect *g*_m_ (Evans *et al.*, 1994; Kogami *et al.*, 2001; Terashima *et al.*, 2001). Differences in leaf anatomical traits and chloroplast ultrastructure have been reported for *D. antarctica* growing along the Maritime Antarctica (Jellings *et al.*, 1983; Gielwanowska and Szczuka, 2005). Regarding *C. quitensis*, plants growing at higher latitudes have smaller and thicker leaves than those grown at lower latitudes, evidenced by a smaller leaf cross-section, with higher mesophyll thickness, narrower adaxial surface, and reduced epidermis thickness (Cavieres *et al.*, 2016). Although these changes have been related to plastic responses to the Antarctic climate (Romero *et al.*, 1999), it is not clear to what extent they may affect the photosynthetic performance of these plants. More specifically, do ultrastructural mesophyll traits induce changes in *g*_m_ and other photosynthetic parameters affecting the carbon assimilation capacity in these Antarctic plant species?

The main objective of the present study was to evaluate *in situ* the photosynthetic performance of two natural populations of *D. antarctica* and *C. quitensis* located at different latitudes within the Maritime Antarctica. In particular, we analyzed the response of photosynthesis to varying measurement temperatures and the diffusive and biochemical constraints to CO_2_ assimilation. To the best of our knowledge, this is the first field study thoroughly analyzing the responses and limitations of photosynthesis on these species.

## Materials and methods

### Study area and plant material

**Fig. 1.**
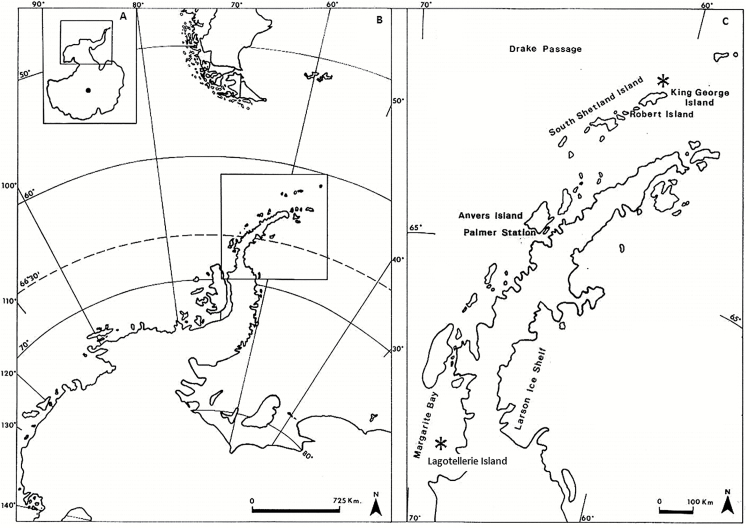
The Antarctic continent (A), Antarctic Peninsula (B, inset from A), South Shetland Islands, and area of the Antarctic Peninsula (C, inset from B). Vascular plants from 62° to 67° South latitude, including the two study areas represented by an asterisk (*): King George Island (62°09'S, 58°28ʹW) and Lagotellerie Island (67°53ʹ20ʺS, 67°25ʹ30ʺW), Marguerite Bay (modified from Alberdi *et al.*, 2002).

Two study sites were selected (Fig. 1): one located in King George Island (KGI), near to Henryk Arctowski Polish Antarctic Station (62°09'S, 58°28'W), and the other in Lagotellerie Island (LAG), in Marguerite Bay (67°53^20ʺS, 67°25ʹ30ʺW). These sites were selected based on the visual similarity of the vegetation, exposure, and elevation, as well as their distance from the coast. The sites are characterized by wide open and relatively flat areas (KGI) or terraces (LAG) covered by vegetation. In both sites, individuals of *D. antarctica* and *C. quitensis* (see Supplementary Fig. S1 at *JXB* online) were randomly selected, within an area of ~300 m^2^, for photosynthesis measurements and sampling of leaves during six sunny days in late January 2015 in LAG and during February 2015 in KGI.

### Climate

Precipitation in both sites falls mainly as snow. Annual precipitation, which occurs mostly during summer, is higher in the northernmost population (~1249 mm), while in Marguerite Bay it is ~360 mm (Turner *et al.*, 2002). During summer, the day length in KGI is ~15 h, and the mean air temperature is 0.7 °C, with average maximum and minimum air temperature of 2.9 °C and –4.1 °C, respectively. In LAG, summer day length is ~17 h, with mean air temperature of –3.5 °C and average maximum and minimum of 1 °C and –10.9 °C, respectively (Utah State University Database; https://climate.usurf.usu.edu).

**Fig. 2.**
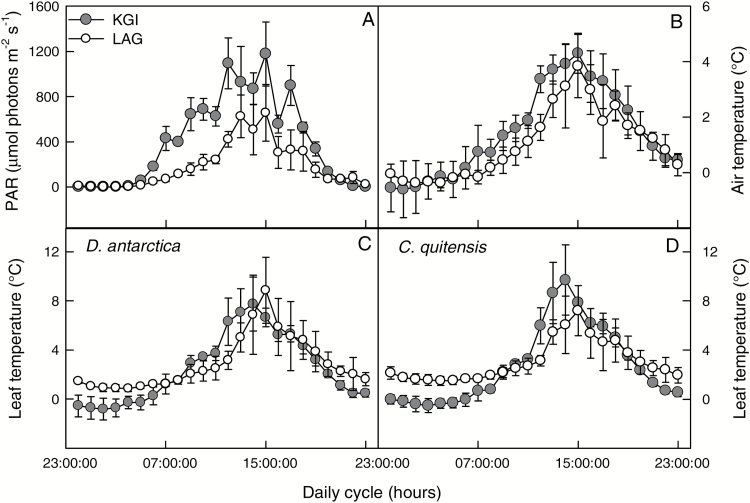
Diurnal course of photosynthetically active radiation (A), air temperature (B), and leaf temperature in *D. antarctica* (C) and *C. quitensis* (D) in King George (KGI) and Lagotellerie Island (LAG) between 17 and 22 January 17 2015. Values are means ± SE

Daily microclimatic records of air temperature at 10 cm above ground level, leaf temperature, and photosynthetically active radiation (PAR) were taken during 6 d in January 2015 (Fig. 2). According to these data, the daily integral of photosynthetic solar radiation in KGI was 30% greater than in LAG (Fig. 2A). Air temperature at KGI tended to be higher than at LAG during most of the daylight period (Fig. 2B). At both locations, leaf temperature of *D. antarctica* and *C. quitensis* was at least 2 °C above ambient temperature, reaching a maximum of ~10 °C (Fig. 2). Differences in leaf temperature between locations were evident during the night, where individuals of both species growing in LAG showed higher leaf temperature (~2 °C) compared with individuals from KGI (Fig. 2C, D). In contrast, during the day, there was a trend for higher leaf temperatures in KGI compared with LAG.

### Leaf gas exchange and chlorophyll fluorescence

Instantaneous gas exchange and Chl *a* fluorescence measurements (Li-6400XT, Li-6400-40 leaf chamber, LI-COR Inc., Lincoln, NE, USA) were performed on a group of leaves (from a branch of *C. quitensis* or a tiller of *D. antarctica*), trying to cover all the IRGA’s chamber area and avoiding leaf overlap. When the leaf area was lower than the chamber area, a correction by actual leaf area inside the chamber was performed.

The response of net photosynthesis CO_2_ uptake (*A*_N_) to varying substomatal CO_2_ concentration (*C*_i_) was studied with the so-called *A*_N_–C_i_ curves. All measurements were made at 1000 µmol photons m^–2^ s^–1^, air relative humidity of 40–50%, and at two leaf temperatures: 10 ºC and 15 ºC. These measurement temperatures corresponded to the maximal leaf temperatures recorded in the field (Fig. 2), and the average of optimal temperature for photosynthesis determined for these species in both populations, respectively (data not shown).

The *A*_N_–*C*_i_ curves were initiated by allowing the leaf to reach steady state (typically 20–30 min after clamping the leaf). Thereafter, *A*_N_–*C*_i_ curves were obtained at 11 different ambient CO_2_ concentrations (*C*_a_s) from 0 to 2000 μmol CO_2_ mol^–1^. Leaves were left to equilibrate at least 5 min at each CO_2_ concentration. Dark mitochondrial leaf respiration (*R*_dark_) was obtained at pre-dawn at a *C*_a_ of 400 μmol CO_2_ mol^–1^ and the two measuring temperatures (10 ºC and 15 ºC). Leaf temperature was measured with a fine-wire thermocouple touching the abaxial surface for the group of leaves. Corrections for the leakage of CO_2_ into and out of the leaf chamber of the Li-6400 were applied to all gas exchange data, as described by Flexas *et al.* (2007).

The quantum efficiency of PSII-driven electron transport was determined using the equation: φ_PSII_=(*F* '_m_–*F*_s_)/*F* '_m_ where *F*_s_ is the steady-state fluorescence in the light (PPFD 1000 μmol quanta m^–2^ s^–1^) and *F* '_m_ the maximum fluorescence obtained with a light-saturating pulse (8000 μmol quanta m^–2^ s^–1^). As φ_PSII_ represents the number of electrons transferred per photon absorbed by PSII, the electron transport rate (ETR) can be calculated as: ETR=φ_PSII_×PPFD αβ, where PPFD is the photosynthetic photon flux density, α is the leaf absorptance, and β is the distribution of absorbed energy between the two photosystems, assumed to be 0.5. The leaf absorptance was directly measured in field-collected plants using a chlorophyll fluorescence system Imaging mini-PAM (Walz, Effeltrich, Germany). Successive illumination of the samples with red (R) and near infrared (NIR) light and the capture of each remission image allowed the calculation of leaf absorptance as follows: Abs=1–R/NIR. Leaf absorptance values were not significantly different between populations, with average values of 0.729 ± 0.02 for *D. antarctica* and 0.738 ± 0.02 for *C. quitensis* (mean ± SE, *n*=50).

The ratio of the electron transport rate and gross photosynthesis (ETR/*A*_G_) was calculated at a *C*_a_ of 400 μmol CO_2_ mol^–1^ air. Gross photosynthesis (*A*_G_) was calculated from the sum of the net CO_2_ assimilation rate (*A*_N_) and half of the mitochondrial respiration in the dark (*R*_dark_).

### Estimation of *g*_m_ and *C*_c_

Mesophyll conductance for CO_2_ (*g*_m_) was calculated from both combined gas-exchange and Chl *a* fluorescence measurements, and anatomical modeling. From the combined gas-exchange and Chl *a* fluorescence measurements, *g*_m_ was calculated as in Harley *et al.* (1992): *g*_m_=*A*_N_/<*C*_i_–{Γ*[ETR+8 (*A*_N_+*R*_L_)]/[ETR–4 (*A*_N_+*R*_L_)]}>, where *A*_N_ and *C*_i_ were obtained from gas exchange measurements at saturating light. The non-photorespiratory CO_2_ evolution rate in the light (*R*_L_) was assumed to be half of *R*_dark_, and the chloroplast CO_2_ compensation point (Γ*) was calculated according to Brooks and Farquhar (1985) from the Rubisco specificity factor (*S*_c/o_) measured *in vitro*. Determination of *g*_m_ was used to calculate the chloroplast CO_2_ concentration (*C*_c_), converting *A*_N_–*C*_i_ curves into A_N_–*C*_c_ curves, as *C*_c_=*C*_i_ –(*A*_N_/*g*_m_).

The maximum velocity of carboxylation (*V*_cmax_) was derived from *A*_N_–*C*_c_ curves according to Farquhar *et al.* (1980) and using the kinetic constants for Rubisco determined for these species at the measurement temperatures (see below).

The approach of Tomás *et al.* (2013) was used for anatomical modeling of *g*_m_. In the field, the central portions of leaves were fixed in formaldehyde, acetic acid, and ethanol, and 4% glutaraldehyde for optical and transmission electron microscopy (JEM1200 EXII, Japan), respectively. Six to 10 micrographs were randomly selected to measure the mesophyll thickness; the mesophyll area exposed to the intercellular air space (*S*_m_) to total leaf surface (*S*) area ratio (*S*_m_/*S*); the chloroplast-exposed surface area to total surface area ratio (*S*_c_/*S*); the chloroplast length (*L*_chl_); the chloroplast thickness (*T*_chl_); and the cell wall thickness (*T*_cw_). All images were analyzed with image analysis software (ImageJ; Wayne Rasband/NIH, Bethesda, MD, USA). The one-dimensional gas diffusion model of Niinemets and Reichstein (2003) was employed to estimate the different leaf anatomical characteristics determining *g*_m_. The determinants of *g*_m_ were divided between gas-phase conductance and the different components of the cellular phase conductances: the cell wall (*l*_cw_), the plasmalemma (*l*_pl_), and inside the cells through the cytosolic path (*l*_cel,tot_).

### Rubisco kinetic characterization at varying temperature

The Rubisco Michaelis–Menten constant for CO_2_ under 21% O_2_ (*K*_c_^air^) was determined in crude extracts obtained as detailed in Galmés et al. (2014*a*). Replicate measurements (*n*=3) were made using independent protein preparations from different individuals. To obtain the Rubisco carboxylase specific activity (*k*_cat_^c^), the maximum rate of carboxylation was extrapolated from the Michaelis–Menten fitted curve and divided by the number of Rubisco active sites in solution, quantified by [^14^C]CABP (2′-carboxyarabinitol-1, 5-bisphosphate) binding (Yokota and Canvin, 1985) as described in Galmés *et al.* (2013). The carboxylase catalytic efficiency was obtained as the ratio *k*_cat_^c^/*K*_c_^air^.

### Determination of the Rubisco specificity for CO_2_/O_2_ (*S*_c/o_) at varying temperature

The Rubisco CO_2_/O_2_ specificity (*S*_c/o_) was measured on purified extracts as in Gago *et al.* (2013), except that values were not normalized to those of wheat Rubisco. Measurements were performed at 5, 15, and 25 ºC, with 3–6 replicates per species and per assayed temperature. For comparative purposes, all Rubisco kinetic traits, including *S*_c/o_, were also determined in wheat (*Triticum aestivum* ‘Cajerne’) at 25 ºC.

For all Rubisco assays, the pH of the assay buffers was accurately adjusted at each measurement temperature. The concentration of CO_2_ in solution in equilibrium with HCO_3_^–^ was calculated assuming a p*K*_a_ for carbonic acid of 6.31, 6.19, and 6.11 at 5, 15, and 25 ºC, respectively. The concentration of O_2_ in solution was assumed to be 400.5, 316.4, and 258.9 nmol ml^–1^ at 5, 15, and 25 ºC, respectively (Truesdale and Downing, 1954).

### Temperature response of the Rubisco kinetic constants

The temperature response of the Rubisco kinetic parameters was fitted for each individual temperature response data set by an Arrhenius-type temperature response function:

$$\text{Parameter}=\text{exp}(c–\Delta H_{\text{a}}/RT)$$ (1)

where *c* is the scaling constant for the parameter, Δ*H*_a_ (J mol^−1^) is the activation energy, *R* is the universal gas constant (8.314 J mol^−1^ K^−1^), and *T* (K) is the temperature. Equation 1 was fitted to the data by iteratively minimizing the sum of squares between the measured and predicted values of each kinetic parameter using the Microsoft Excel Solver function.

### Statistical analyses

Fully factorial two-way ANOVAs were performed on each species to assess differences between populations and measurement temperatures. Differences between means were assessed by *a posteriori* Tukey test (*P*<0.05). These analyses were performed with the SPSS statistics 19.0 software package (IBM-software, New York, USA). A Pearson correlation analysis was performed to assess the relationship between the anatomical derived *g*_m_ and studied anatomical traits. Goodness of fit to saturation curves was assessed in: *A*_N_/*g*_tot_, *A*_N_/*C*_c_, and *A*_N_/*g*_m_. All these analyses were done in Statistica 7.0 (Stat Soft Inc. Tulsa, OK, USA).

## Results

### Leaf carbon exchange in Antarctic plants at different measurement temperatures

**Fig. 3.**
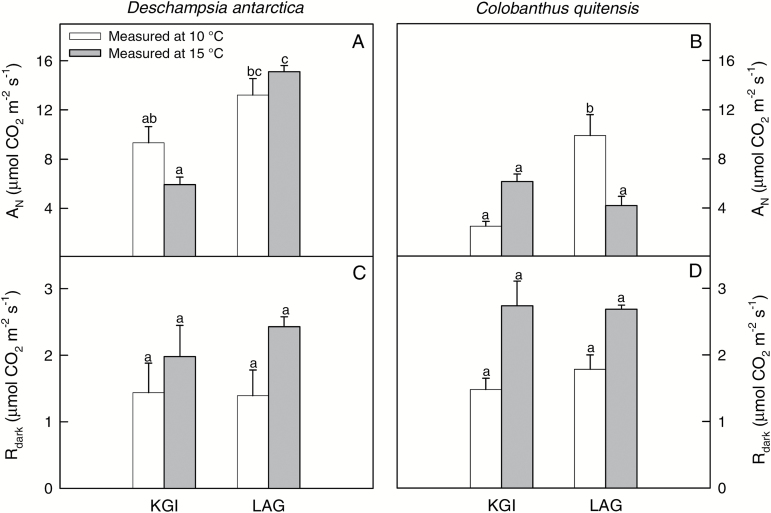
The net photosynthetic CO_2_ assimilation rate (*A*_N_) and dark respiration (*R*_dark_) of *D. antarctica* (A, C) and *C. quitensis* (B, D) in King George (KGI) and Lagotellerie Island (LAG), measured at 10 °C (white bars) or 15 °C (gray bars). Values are means ± SE (*n*=5–7). Different letters indicate statistically significant differences for each species between populations and measurement temperature according to Tukey (*P*<0.05).

Plants from LAG exhibited higher net CO_2_ assimilation rates (*A*_N_) at ambient CO_2_ concentration than those from KGI, with no differences between measurement temperatures in the studied populations, although in both species the interaction between these two factors was significant (Supplementary Table S1; Supplementary Fig. S2). In *D. antarctica*, *A*_N_ was higher in LAG at 15 °C (15.11 ± 0.51 µmol CO_2_ m^–2^ s^–1^) compared with KGI (5.93 ± 0.60 µmol CO_2_ m^–2^ s^–1^; Fig. 3A), but *A*_N_ was similar across the two sites at 10 °C (~12 µmol CO_2_ m^–2^ s^–1^). In *C. quitensis*, the highest *A*_N_ value was observed in LAG at 10 °C (9.88 ± 1.71 µmol CO_2_ m^–2^ s^–1^) and the lowest in KGI at 10 ºC (2.53 ± 0.37 µmol CO_2_ m^–2^ s^–1^; Fig. 3B). There were no differences in the dark respiration rate (*R*_dark_) between populations or measurement temperatures (Fig. 3C, D; Supplementary Table S1). This indicates that the observed differences in *A*_N_ were not due to differences in *R*_dark_, but to differences in the photosynthetic process and its determinants.

### Diffusive limitations to photosynthesis

**Fig. 4.**
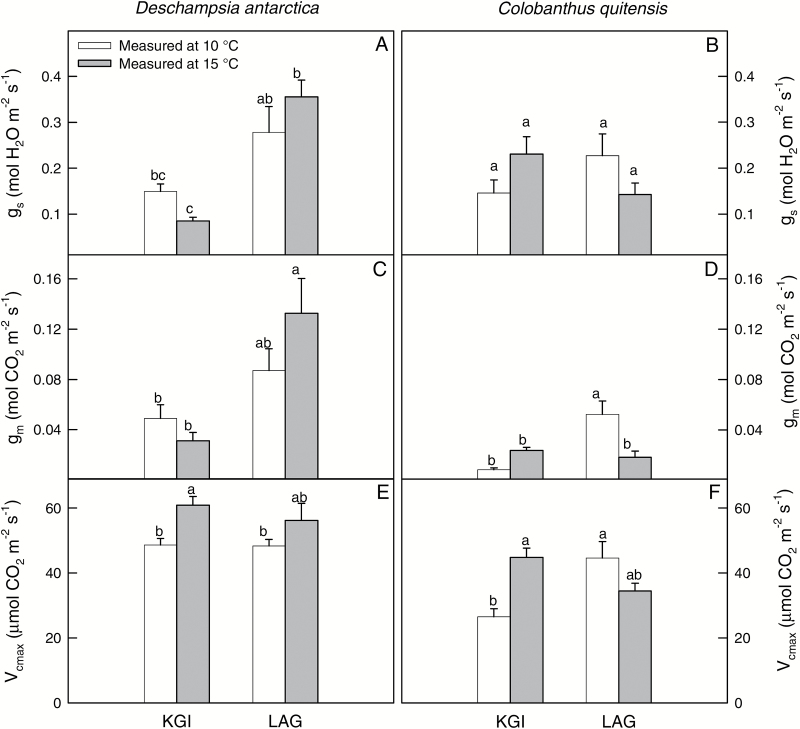
The stomatal (A, B) and the leaf mesophyll (C, D) conductances, and the maximum rate of Rubisco carboxylation (E, F) of *D. antarctica* (left) and *C. quitensis* (right) in King George (KGI) and Lagotellerie Island (LAG), measured at 10 °C (white bars) or 15 °C (gray bars). Values are means ± SE (*n*=5–7). Different letters indicate statistically significant differences between populations for each species and measurement temperature according to Tukey (*P*<0.05).

**Fig. 5.**
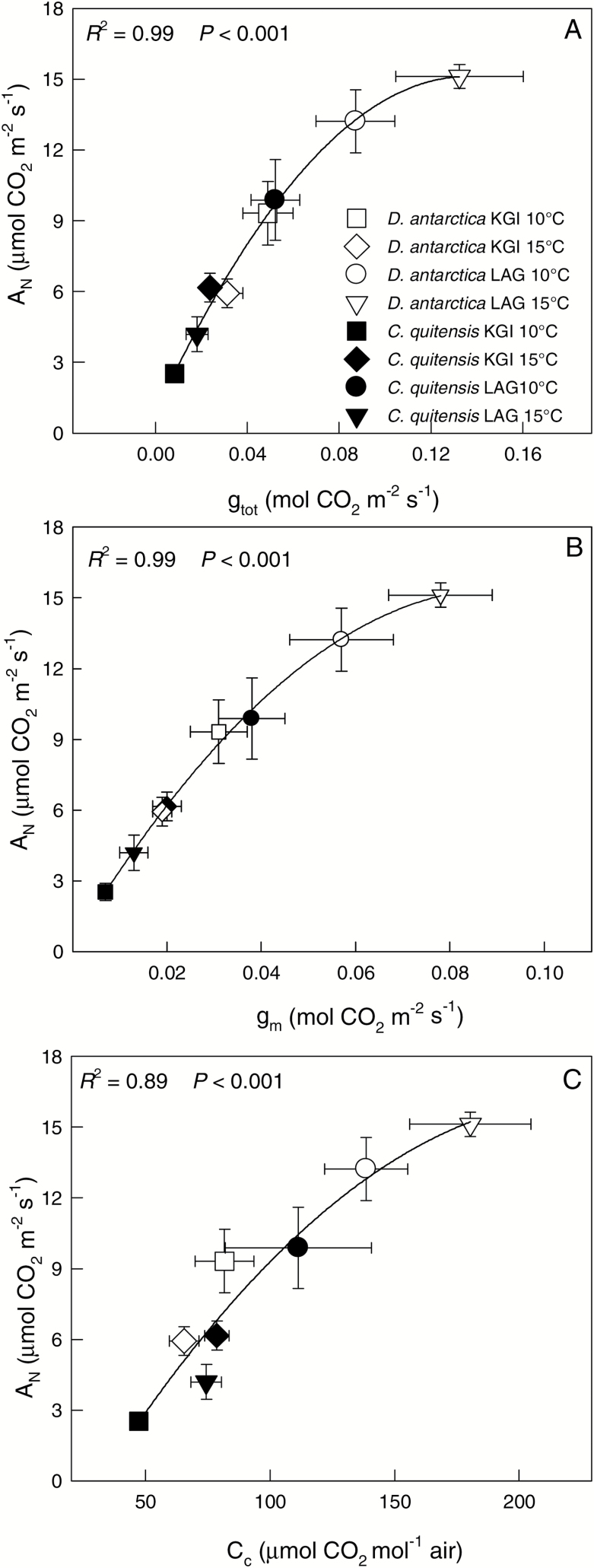
The relationship of (A) the total leaf conductance (*g*_tot_), (B) the leaf mesophyll conductance (*g*_m_), and (C) the chloroplast CO_2_ concentration (*C*_c_) to the net photosynthetic CO_2_ assimilation rate (*A*_N_) of *D. antarctica* (open circles) and *C. quitensis* (filled circles) in King George (KGI) and Lagotellerie Island (LAG). Goodness of fit with a saturation model is shown for both species and considering all populations and measurement temperatures together. Values are means ± SE (*n*=5–7).

Stomatal (*g*_s_) and leaf mesophyll (*g*_m_) conductances for both species showed similar trends to those described for *A*_N_ (Fig. 4; Supplementary Table S1). Values of *g*_s_ for *D. antarctica* ranged between 0.08 ± 0.01 mol H_2_O m^–2^ s^–1^ at KGI at 15 ºC and 0.35 ± 0.03 mol H_2_O m^–2^ s^–1^ in LAG at 15 ºC. For *C. quitensis*, *g*_s_ varied from 0.14 ± 0.02 mol H_2_O m^–2^ s^–1^ in LAG at 15 ºC to 0.23 ± 0.03 mol H_2_O m^–2^ s^–1^ in KGI at 15 ºC (Fig. 4A, B). Estimated *g*_m_ values were lower than those of *g*_s_, with the minimum value estimated in *C. quitensis* from KGI at 10 ºC (0.01 ± 0.01 mol CO_2_ m^–2^ s^–1^), and the highest in *D. antarctica* from LAG at 15 ºC (0.13 ± 0.02 mol CO_2_ m^–2^ s^–1^). The low *g*_m_ determined low total leaf conductance to CO_2_ (*g*_tot_), which significantly correlated with *A*_N_ (Fig. 5A). This indicates that the photosynthetic rates in the Antarctic vascular plants under field conditions were limited by diffusional components in general, and low *g*_m_ in particular (Fig. 5B). The good correspondence between *A*_N_ and the chloroplastic CO_2_ concentration (*C*_c_) further confirms that carbon fixation in these species was constrained by low availability of CO_2_ at the carboxylation sites (Fig. 5C). In general, diffusion limitations to photosynthesis were more evident in *C. quitensis* (Figs 4, 5), which is consistent with the lowest CO_2_ assimilation rates found in this species (Fig. 3B).

### Mesophyll conductance modeled from leaf anatomy

The studied populations of *D. antarctica* showed similar leaf anatomy, with the exception of the mesophyll thickness (*T*_mes_), which was higher in plants from LAG, and the chloroplast surface area facing intercellular air spaces per leaf area (*S*_c_/*S*), which was larger in plants from KGI (Table 1). No differences between populations were found in ultrastructural leaf characteristics (Supplementary Fig. S3) such as cell wall thickness (*T*_cw_), average distance between chloroplasts (Δ*L*_cyt_), and their size (*L*_chl_ and *T*_chl_). In addition, large numbers of organelles around chloroplasts were observed in both plant species (Supplementary Figs S3, S4). For *C. quitensis*, there were differences between populations in most of the anatomical parameters evaluated, except in *T*_mes_ and the chloroplast length (*L*_chl_) (Table 1). In particular, *C. quitensis* plants from KGI exhibited higher *T*_cw_, Δ*L*_cyt_, and chloroplast thickness (*T*_chl_) than plants from LAG (Table 1). The contrasting anatomical traits between both populations of *C. quitensis* determined differences in *g*_m_ modeled from leaf anatomy (0.013 ± 0.001 mol CO_2_ m^–2^ s^–1^ in KGI versus 0.039 ± 0.004 mol CO_2_ m^–2^ s^–1^ in LAG).

**Table 1.** The mesophyll thickness between the two epidermal layers (*T*_mes_), the cell wall thickness (*T*_cw_), the average distance between chloroplasts (Δ*L*_cyt_), the chloroplast thickness (*T*_chl_), the chloroplast length (*L*_chl_), the CO_2_ transfer conductances across the intercellular air space (*g*_ias_), the liquid phase (*g*_liq_), and the mesophyll conductance for CO_2_ (*g*_m_) calculated from leaf anatomical measurements, the mesophyll (*S*_m_/*S*) and chloroplast (*S*_c/_*S*) surface area facing intercellular air spaces per leaf area, for *D. antarctica* and *C. quitensis* from King George (KGI) and Lagotellerie Island (LAG)

	*D. antarctica*		*C. quitensis*	
	KGI	LAG	KGI	LAG
*T*_mes_ (μm)	101.03 ± 5.43 a	136.27 ± 5.14 b	322.29 ± 13.42 a	290.77 ± 18.91 a
*T*_cw_ (µm)	0.26 ± 0.01 a	0.22 ± 0.03 a	0.35 ± 0.06 b	0.21 ± 0.02 a
Δ*L*_cyt_ (µm)	0.68 ± 0.18 a	0.3 ± 0.16 a	0.53 ± 0.06 b	0.05 ± 0.03 a
*T*_chl_ (µm)	2.96 ± 0.24 a	3.58 ± 0.52 a	5.12 ± 0.66 b	1.64 ± 0.12 a
*L*_chl_ (µm)	4.40 ± 0.36 a	5.48 ± 0.56 a	5.32 ± 0.28 a	5.92 ± 0.36 a
*g*_ias_ (m s^–1^)	0.046 ± 0.006 a	0.031 ± 0.004 a	0.013 ± 0.001 a	0.019 ± 0.001 b
*g*_liq_ (m s^–1^)	0.0005 ± 0.0001 a	0.0005 ± 0.0001 a	0.0003 ± 0.0000 a	0.0001 ± 0.0001 b
*g*_m_ (mol m^–2^ s^–1^)	0.022 ± 0.002 a	0.023 ± 0.004 a	0.013 ± 0.001 a	0.039 ± 0.004 b
*S*_m_/*S* (m^2^ m^–2^)	6.40 ± 0.75 a	6.28 ± 0.51 a	4.33 ± 0.48 a	8.56 ± 0.53 b
*S*_c_/*S* (m^2^ m^–2^)	2.24 ± 0.48 b	1.16 ± 0.08 a	2.06 ± 0.32 a	4.42 ± 0.63 b

Values are means ± SE (*n*=6–10).

Different letters indicate statistically significant differences between populations for each species (*P*<0.05).

In general, anatomy-based *g*_m_ values were lower than those estimated *in vivo* by the method of Harley *et al.* (1992) (Table 1). It should be noted that anatomical measurements do not consider the different leaf temperatures recorded between populations (Fig. 2); therefore, the comparison with the ‘Harley’ *g*_m_ should be treated with caution. Despite this limitation, some of the described trends for *g*_m_ estimated by the method of Harley *et al.* (1992) were also found with the anatomy-based *g*_m_ (compare Fig. 4D with Table 1). For example, *C. quitensis* from LAG at 10 ºC showed higher values compared with the KGI. In contrast, the higher ‘Harley’ *g*_m_ found in *D. antarctica* from LAG compared with KGI (Fig. 4C) was not supported by the anatomical modeling of *g*_m_ (Table 1).

**Fig. 6.**
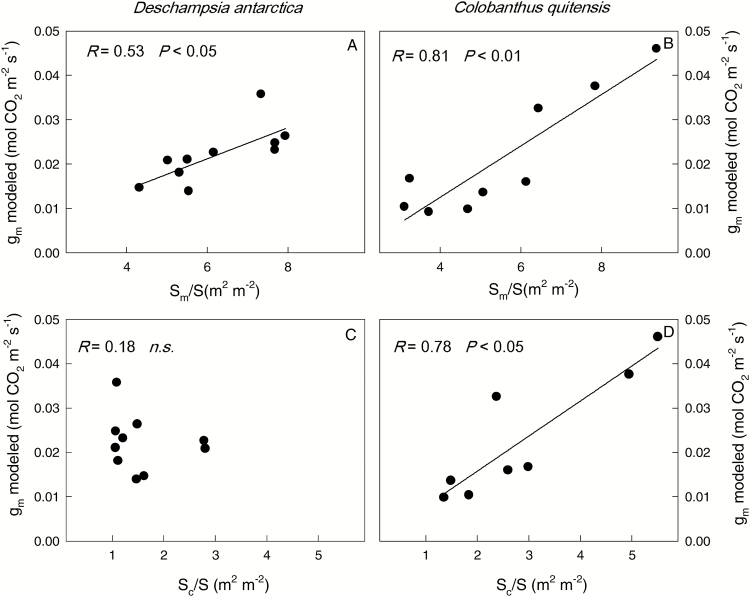
Correlations of the leaf mesophyll conductance modeled with anatomical parameters (*g*_m_ modeled) with the mesophyll (*S*_m_/*S*) and chloroplast (S_c/_S) surface area facing intercellular air spaces per leaf area in *D. antarctica* (left) and *C. quitensis* (right). The Pearson correlation coefficient and the significance of the relationship are shown for each species considering both populations together. Values are means ± SE (*n*=3–5).

In *C. quitensis*, the mesophyll (*S*_m_/*S*) and chloroplast (*S*_c_/*S*) surface areas facing the intercellular air spaces per leaf area were significantly higher in LAG compared with KGI (Table 1), and the values of *S*_m_/*S* and *S*_c_/*S* correlated positively with *g*_m_ modeled from leaf anatomy (Fig. 6). In *D. antarctica*, *S*_c_/*S* but not *S*_m_/*S* differed between populations, and only *S*_m_/*S* but not *S*_c_/*S* correlated with *g*_m_ (Fig. 6).

In both species, the CO_2_ transfer conductance across the liquid phase (*g*_liq_) was lower than that across the intercellular air space (*g*_ias_) (Table 1), which yielded a low quantitative *g*_m_ limitation by the intercellular air spaces (*l*_ias_) (Table 2). Regarding the components of *g*_liq_: the cell wall (*l*_cw_), but mainly the cytoplasm and stroma (*l*_cet,tot_) limitations determined the low internal diffusion of CO_2_ observed in the Antarctic vascular plants.

**Table 2.** Quantitative limitation analysis of the leaf mesophyll conductance to CO_2_ (*g*_m_) for *D. antarctica* and *C. quitensis* from King George (KGI) and Lagotellerie Island (LAG) due to different anatomical components of the diffusion pathway: intercellular spaces (*l*_ias_), cell wall (*l*_cw_), plasmalemma (*l*_pl_), and inside the cell (*l*_cel, tot_)

	*D. antarctica*		*C. quitensis*	
	KGI	LAG	KGI	LAG
*l*_ias_ (%)	1.1 ± 0.1 a	1.7 ± 0.2 a	2.5 ± 0.4 a	4.6 ± 0.1 b
*l*_cw_ (%)	34.4 ± 1.3 a	27.4 ± 2.1 b	35.4 ± 5.4 a	49.5 ± 6.2 a
*l*_pl_ (%)	2.3 ± 0.1 a	2.3 ± 0.2 a	2.0 ± 0.3 a	3.3 ± 0.1 b
*l*_cel, tot_ (%)	62.3 ± 1.5 a	68.4 ± 2.1 a	59.8 ± 5.4 a	42.5 ± 6.1 b

Values are means ± SE (*n*=4–10).

Different letters indicate statistically significant differences between populations for each species (*P*<0.05).

### Biochemical determinants of photosynthesis

The maximum rate of Rubisco carboxylation (*V*_cmax_) increased with the measurement temperature in both plant species from KGI, but non-significant differences between measurement temperatures were found in plants from LAG (Fig. 4E, F). For all the range of *A*_N_–*C*_c_ curves, *A*_N_ was linearly correlated to *C*_c_ (Supplementary Fig. S5), which impeded the estimation of the maximum rate of electron transport (*J*_max_).

**Fig. 7.**
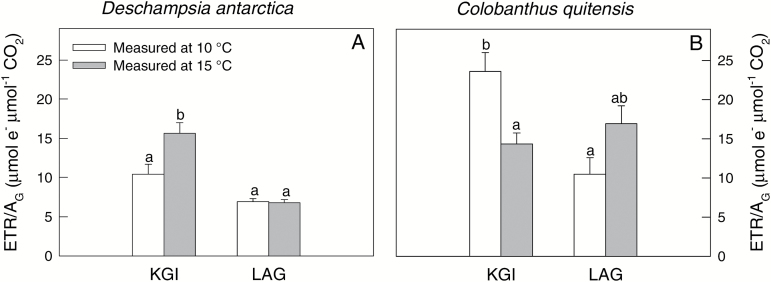
The ratio of the electron transport rate and gross photosynthesis (ETR/*A*_G_) for *D. antarctica* (A) and *C. quitensis* (B) in King George (KGI) and Lagotellerie Island (LAG), measured at 10 °C (white bars) or 15 °C (gray bars). Values are means ± SE (*n*=5–7). Different letters indicate statistically significant differences for each species between populations and measurement temperatures according to Tukey (*P*<0.05).

The ratio between the electron transport rate and gross CO_2_ assimilation rate (ETR/*A*_G_) which is used as a proxy of the amount of reducing power per unit of fixed CO_2_ (Flexas *et al.*, 2002), varied between measurement temperatures and populations in both species (Fig. 7). In plants from KGI, the increase in the measurement temperature induced an increase in ETR/*A*_G_ in *D. antarctica* but a decrease in *C. quitensis*. In plants from LAG, there were no temperature-driven changes in ETR/*A*_G_ in either of the two species. The relationship between ETR/*A*_G_ and *C*_c_ was highly significant and negative (Supplementary Fig. S6), indicative of enhanced photorespiration rates under low CO_2_ availability. Except for KGI at 15 ºC, *C. quitensis* exhibited higher values for ETR/*A*_G_—consistent with lower values for *C*_c_.

### Rubisco *in vitro* kinetic parameters and their temperature dependence

The affinity of Rubisco for CO_2_ measured as *K*_c_^air^ at 25 ºC was 18.7 ± 0.4 µM and 23.4 ± 1.8 µM for *C. quitensis* and *D. antarctica*, respectively (Table 3). The maximum rate of the carboxylase catalytic turnover (*k*_cat_^c^) was 3.76 ± 0.29 s^–1^ in *C. quitensis* and 4.10 ± 0.75 s^–1^ in *D. antarctica*. The carboxylase catalytic efficiency assessed by the *k*_cat_^c^/*K*_c_^air^ ratio ranged between 0.18 s^–1^ µM^–1^ and 0.20 s^–1^ µM^–1^ in both Antarctic species. At 25 ºC, the values of the Rubisco specificity factor (*S*_c/o_) were 99.5 ± 4.8 mol mol^–1^ in *D. antarctica* and 97.1 ± 2.4 mol mol^–1^ in *C. quitensis* (Table 3).

**Table 3.** *In vitro* Rubisco kinetic parameters and their temperature response in *D. antarctica* and *C. quitensis* The Rubisco Michaelis–Menten constant affinity for CO_2_ under 21% O_2_ (*K*_c_^air^), the maximum carboxylase catalytic turnover rate (*k*_cat_^c^), the carboxylase catalytic efficiency (*k*_cat_^c^/*K*_c_^air^), and the specificity factor (*S*_c/o_). For each parameter, the scaling constant (*c*) and the activation energy (Δ*H*_a_) are shown.

Assay temperature	*D. antarctica*				*C. quitensis*			
	*K*_c_^air^ (µM)	*k*_cat_^c^ (s^–1^)	*k*_cat_^c^/*K*_c_^air^ (s^–1^ µM^–1^)	*S*_c/o_ (mol mol^–1^)	*K*_c_^air^ (µM)	*k*_cat_^c^ (s^–1^)	*k*_cat_^c^/*K*_c_^air^ (s^–1^ µM^–1^)	*S*_c/o_ (mol mol^–1^)
5 ºC	10.9 ± 1.2 a*	0.4 ± 0.1 a*	0.04 ± 0.00 a	167.5 ± 2.9 c	6.9 ± 0.4 a*	0.2 ± 0.1 a*	0.03 ± 0.00 a	172.4 ± 2.0 c
15 ºC	14.7 ± 0.8 a*	1.4 ± 0.2 a	0.09 ± 0.01 ab	151.3 ± 4.5 b*	11.1 ± 0.6 b*	1.1 ± 0.1 b	0.10 ± 0.01 b	129.8 ± 3.7 b*
25 ºC	23.4 ± 1.8 b	4.1 ± 0.8b	0.18 ± 0.04 b	99.5 ± 4.8 a	18.7 ± 0.4 c	3.8 ± 0.3 c	0.20 ± 0.01 c	97.1 ± 2.4 a
*c*	14.5 ± 1.8	33.2 ± 0.8	18.4 ± 2.5	–1.7 ± 0.6	17.1 ± 0.7	41.0 ± 2.7	20.9 ± 0.2	–3.2 ± 0.5
Δ*H*_a_ (kJ mol^–1^)	28.2 ± 4.4	78.8 ± 1.5*	49.9 ± 5.6	–15.9 ± 1.4	35.2 ± 1.6	98.3 ± 6.6*	55.6 ± 0.5	–19.3 ± 1.2

Different letters indicate statistically significant differences among assay temperatures in each species, and an asterisk denotes statistically significant differences between *D. antarctica* and *C. quitensis* for each assay temperature (*P*<0.05).

Values are means ± SE (*n*=3–6).

For comparative purposes, we also measured the kinetic parameters at 25 ºC in Rubisco from wheat (*Triticum aesticum* ‘Cajerne’). The values were as follows: *K*_c_^air^=16.0 µM; *k*_cat_^c^=2.2 s^−1^; *k*_cat_^c^/*K*_c_^air^=0.14 s^–1^ µM^–1^; *S*_c/o_=101.1 mol mol^–1^.

Differences were observed in the temperature response of Rubisco between *D. antarctica* and *C. quitensis* (Table 3). For instance, *D. antarctica* presented higher values for *K*_c_^air^ than *C. quitensis* at 5 °C and 15 °C. Differences in *k*_cat_^c^ between *D. antarctica* and *C. quitensis* were found only at 5 °C. In consequence, the Rubisco carboxylase catalytic efficiency did not differ between species at any of the assayed temperatures. Regarding *S*_c/o_, differences between the two Antarctic plant species were observed only at 15 ºC, where *D. antarctica* presented higher values than *C. quitensis* (*P*<0.05).

The differences in the temperature response of Rubisco resulted in a trend for higher absolute values of the energy of activation (Δ*H*_a_) of the different kinetic parameters in *C. quitensis*, although significant differences between both species were only observed in Δ*H*_a_ of *k*_cat_^c^ (78.8 ± 1.5 kJ mol^–1^ in *D. antarctica* versus 98.3 ± 6.6 kJ mol^–1^ in *C. quitensis*).

## Discussion

The leaf anatomical and biochemical traits described here for the Antarctic vascular plants growing at different latitudes within Antarctica and their implications on the photosynthetic performance of these species constitute new insights into the plant functional responses to cold conditions. While ultrastructural traits of the leaf mesophyll restricted CO_2_ transfer and limited the photosynthetic capacity of the two Antarctic vascular plants, the kinetic traits of Rubisco, characterized by high affinity for CO_2_ and relative high values for *k*_cat_^c^, seem crucial to optimize carbon assimilation despite the restrictions of CO_2_ transport inside the leaf. The former suggests an important functional adaptation that, together with other traits (Cavieres *et al.*, 2016), allows *D. antarctica* and *C. quitensis* to survive and grow in the harsh climate conditions of the Antarctica. As these two Antarctic species differ in their habitat requirements, plant morphology, leaf anatomy, and photosynthetic optimum temperature (Cavieres *et al.*, 2016), an interspecific comparison was not considered here, except for Rubisco kinetic parameters.

### Leaf mesophyll conductance to CO_2_ limits carbon assimilation in Antarctic plants

This is the first study assessing *g*_m_ in Antarctic vascular plants, and how changes in the ultrastructure of the mesophyll affect *g*_m_, and hence the carbon acquisition in these species. According to our results, the range of *g*_m_ values and the *g*_m_/*g*_s_ ratio of these plant species are among the lowest reported so far for higher plant species (De Lucía et al., 2003; Flexas *et al.*, 2009, 2014; Tomás *et al.*, 2013; Peguero-Pina *et al.*, 2015*a*). Under certain conditions, *g*_m_ can be the most significant photosynthetic limitation (Flexas *et al.*, 2012; Tomás *et al.*, 2013; Galmés *et al.*, 2014*a*; Niinemets and Keenan, 2014; Carriquí *et al*., 2014; Peguero-Pina *et al.*, 2015a, b). As the photosynthetic rates of the Antarctic vascular plants were highly correlated with *g*_m_ (Fig. 5B), this factor seems to be the main constraint for the photosynthetic process in these plant species in the field. Positive correlations between *A*_N_ and *g*_m_ have been previously reported (e.g. Evans and Loreto, 2000; Warren, 2008), and recent meta-analyses suggest that *g*_m_ is also associated with the structure of leaves, determining the diffusion limitations of photosynthesis (Niinemets and Sack, 2006; Warren, 2008). Thus, our results highlight that morphoanatomical leaf characteristics regulating *g*_m_ are key determinants of the photosynthetic functioning in the two Antarctic vascular plant species.

Large differences in *g*_m_ have been shown both between and within species with different leaf forms and habits (e.g. Flexas *et al.*, 2008; Warren, 2008). The low *g*_m_ found in the Antarctic vascular plants in the field seems to be related to leaf anatomical traits that affect CO_2_ diffusion across the intercellular air space (Table 1), especially with the limitations associated with cell walls, cytoplasm, and stroma (Table 2). The leaf mesophyll diffusion limitations were especially evident in *C. quitensis*, which is consistent with the low photosynthetic rate of this species and with the high values of ETR/*A*_G_ (Fig. 7), indicative of enhanced photorespiration rates. Higher leaf mesophyll thickness is commonly associated with a greater number of leaf mesophyll cell layers (Niinemets *et al.*, 2009). In *C. quitensis*, the highest leaf mesophyll thickness was accompanied by the largest area of leaf mesophyll exposed to intercellular air space (*S*_m_), and thus the greatest *S*_m_ to total leaf surface area ratio (*S*_m_/*S*) (Table 1). Provided that the numbers of chloroplasts are similar in mesophyll cells, a larger *S*_m_/*S* also implies a greater ratio between the chloroplast-exposed surface area and the total surface area (*S*_c_/*S*) (Terashima *et al.*, 2005, 2006). As a larger *S*_c_/*S* implies more parallel pathways for CO_2_ liquid-phase diffusion, *g*_m_ correlates positively with *S*_c_/*S* (Fig. 6). Actually, higher leaf density has been associated with reduced gas-phase volume, and smaller and more densely packed leaf mesophyll cells with thicker cell walls (Niinemets, 1999; Niinemets and Sack, 2006). Such modiﬁcations could reduce the liquid-phase diffusion conductance and g_m_ (Terashima *et al.*, 2005; Evans *et al.*, 2009). In addition, the observed differences in *g*_m_ between both populations of *C. quitensis* can be partially attributed to the variation in other leaf anatomical traits such as *T*_cw_, Δ*L*_cyt_, and *T*_chl_ (Table 1). Specifically, the cell wall and the resistances imposed by the cytoplasm and stroma exerted the highest limitations in the *g*_m_ (Table 2). Further, as was noted above, *S*_m_/*S* and *S*_c_/*S* are two of the most important anatomical traits influencing *g*_m_ (Tosens *et al.*, 2012; Peguero-Pina *et al.*, 2012; Carriquí *et al*., 2014).

Anatomical characteristics could not be the only explanation for the observed differences in *g*_m_, as those were also found between measurement temperatures in plants growing in LAG (Fig. 4C, D). It seems likely that some (not yet fully understood) biochemical components of *g*_m_ could be involved in the regulation of *g*_m_. Among them, carbonic anhydrase (CAs) (Fabre *et al.*, 2007) and aquaporins (AQPs) (Terashima and Ono, 2002) have been shown to modify *g*_m_*in vivo* in response to varying measurement temperatures. The discrepancy between *g*_m_ estimates based on anatomical measurements and those based on conventional gas-exchange methods suggests the existence of these facilitating mechanisms for CO_2_ diffusion. However, to the best of our knowledge, there are no reports on the activity and abundance of these proteins in Antarctic vascular plants, and thus we cannot draw any general conclusion on this issue.

The anatomical features of the Antarctic species have been regarded as adaptive responses to the harsh climate conditions of Antarctica (Cavieres *et al.*, 2016). Among them, the large number of organelles (mitochondria or peroxisomes) around the chloroplasts in both Antarctic species (Supplementary Figs S3, S4) have been suggested as facilitators for the CO_2_ exchange between respiration and photorespiration processes (Gielwanoeska and Szczuka, 2005). In addition, both species have xeromorphic leaf characteristics (Nobel, 1980; Vieira and Mantovani, 1995; Romero *et al.*, 1999), which are related to the water limitations due to the low temperature and strong winds that characterize the Antarctic climate (Smith, 1993). Some features, such as the presence of two bundle sheaths in leaf vascular bundles (mestome), of *D. antarctica* have been associated with an adaptation to high radiation in order to optimize photosynthesis and water use efficiency (WUE) (Pyykkö, 1966). Evert *et al.* (1985) suggested that the mestome functions as a leaf endodermis, limiting apoplastic movement of water across the mesophyll. This trait, along with a high stomatal density and leaf mass area, confer a high capacity to control water loss on *D. antarctica* (Vieira and Mantovani, 1995; Xiong *et al*., 2000; Alberdi *et al.*, 2002). Decreased water movement across the leaf mesophyll could be advantageous to avoid heat loss in cold environments, being an important adaptation for these habitats (Sage, 2001). These characteristics are consistent with the low *g*_m_ values determined in the present study using both gas-exchange/fluorescence and anatomical methods.

The low *g*_m_ values resulted in low *g*_tot_ values, and therefore low *C*_c_. According to our results, *A*_N_ was highly correlated with *g*_m_, *g*_tot_, and *C*_c_ (Fig. 5), confirming the predominant role of leaf CO_2_ diffusion in the photosynthetic performance of Antarctic plant species. In this sense, reduced photosynthesis, due to low *g*_m_, seems to be the penalty of structurally robust leaves that the Antarctic plant species have to pay to survive in extremely stressful conditions.

### Rubisco performance alleviates the low mesophyll conductance

The Rubisco kinetic parameters, and their temperature response, have been related to species differences in the photosynthetic performance under varying conditions (Galmés *et al.*, 2014a, 2015, 2016; Sharwood *et al.*, 2016). The temperature dependence of Rubisco kinetics revealed some differences between the two Antarctic angiosperm species. In particular, the energy of activation (Δ*H*_a_) for *k*_cat_^c^ was higher in *C. quitensis* compared with *D. antarctica*, indicative of higher thermal sensitivity (Table 3). The values for Δ*H*_a_ of *k*_cat_^c^ in the two Antarctic species are among the highest in Streptophyta, and do not support the reported trend (see compilation by Galmés *et al.*, 2015) that the Rubisco enzymes of C_3_ species adapted to cool habitats have a lower plastic response to temperature changes compared with Rubisco of C_3_ species from warm environments. The molecular and biochemical causes of this apparent discrepancy, already observed in other species from cool habitats such as *Atriplex glabriuscula* (Badger and Collatz, 1977), are unknown and should be explored in depth. In contrast, the high values for both *S*_c/o_ and Δ*H*_a_ of *k*_cat_^c^ observed in the Antarctic species are in accordance with the transition state theory of Tcherkez *et al.* (2006).

In fact, the values of *S*_c/o_ measured at 25 ºC in both Antarctic plants (Table 3) are among the highest values reported so far for higher plant species (e.g. Galmés *et al.*, 2005; Orr *et al.*, 2016). We note that for comparative purposes between data sets, we also measured Rubisco kinetic parameters in wheat at 25 ºC (shown in the footnotes of Table 3) and the obtained values were similar to previous data (e.g. Galmés *et al.*, 2005; Prins *et al.*, 2016; Orr *et al.*, 2016). Regarding *K*_c_^air^ at 25 ºC, both Antarctic plants, but in particular *D. antarctica*, presented values comparable with those reported for species adapted to xeric environments (Galmés *et al.*, 2014*a*). Notably, despite their high affinity for CO_2_, Rubisco from the Antarctic plant species retained high *k*_cat_^c^, similar to species closely related to *D. antarctica* such as *Poa arctica* and *Poa pratensis* (Sage, 2002). The high *k*_cat_^c^ leads to values for the carboxylase catalytic efficiency (*k*_cat_^c^/*K*_c_^air^) higher than those reported for species from xeric habitats (Galmés *et al.*, 2014*a*). Further, at 15 ºC (a temperature similar to the maximal diurnal leaf temperature recorded in the field; Fig. 2), Rubisco from both Antarctic plants increased the specificity for CO_2_ to levels similar to that reported for xeromorphic species (Galmés *et al.*, 2005).

Although it has been suggested that the selective pressures for greater CO_2_ affinity for Rubisco have been high in species adapted to high temperature and low soil water availability (Galmés *et al.*, 2005, 2014*a*), there is also evidence that in cold environments the selective pressures may favor Rubisco with higher *k*_cat_^c^ (Sage, 2002; Yamori *et al.*, 2009). Interestingly, the Antarctic species have evolved under both dry and cold conditions, and here we found that their Rubiscos show a high affinity for CO_2_ and retain high *k*_cat_^c^. The high *S*_c/o_ in the Antarctic plants is additional evidence in favor of the hypothesis that CO_2_ limitations shaped the evolution of their Rubisco kinetics (e.g. Raven, 2000; Young *et al.*, 2012; Galmés *et al.*, 2014*b*). In the case of the Antarctic plants, the low availability of CO_2_ at the sites of carboxylation is driven by adaptive anatomical traits to resist the extreme climatic conditions of low temperature and strong winds. It has been demonstrated that under conditions that promote drought, higher *S*_c/o_ reduces ribulose bisphosphate (RuBP) oxygenation and favors the carboxylase reaction (Galmés *et al.*, 2005). Thus, it seems likely that the anatomical features that determine a low CO_2_ diffusion in these species are partially counterbalanced by a highly efficient Rubisco. The concentration of active Rubisco, calculated from *V*_cmax_=*k*_cat_^c^×[active Rubisco sites], was notably high in both Antarctic species at 15 ºC: ~44.57 ± 1.9 µmol m^–2^ and 42.31 ± 2.84 µmol m^–2^ in *D. antarctica* and *C. quitensis*, respectively. Although these data should be confirmed by direct measurements in leaf extracts, they suggest that the decrease in *k*_cat_^c^ at low temperatures is compensated by increased amount of [active Rubisco sites], following trends already reported in other species (e.g. Yamori *et al.*, 2005, 2006).

The high *S*_c/o_ and low *K*_c_^air^, as well as the constitutive low *g*_m_ of Antarctic plants, are not their unique traits, resembling those documented for drought-adapted species. The presence of bundle sheaths found in *D. antarctica* and also in *C. quitensis* (Vieira and Mantovani, 1995) is a characteristic of plants from xeric climates (Sage, 2001). In addition, Montiel *et al.* (1999) reported remarkably high values of instantaneous WUE in *D. antarctica*, more typical of C_4_ and Crassulacean acid metabolism (CAM) species than of C_3_ species. The high WUE of *D. antarctica* is likely to be related to the diffusive and biochemical determinants identified in the present study.

### Concluding remarks

The present study provides new field data on the photosynthetic performance and diffusive and biochemical limitations of the two Antarctic plants. This work incorporates leaf anatomical traits related to CO_2_ assimilation and increases the range of knowledge of the diversity of Rubisco kinetics parameters in two relevant species.

The ultrastructural traits of the leaf mesophyll in field-grown Antarctic plants ultimately restricted the CO_2_ leaf transfer capacity and limited the photosynthetic capacity of these species. Under the cold and dry climate conditions of the Antarctica, the high Rubisco affinity for CO_2_ and relatively high values for *k*_cat_^c^ seem crucial to optimize carbon assimilation. Overall, these results constitute new insights regarding the functional adaptations to highly stressful conditions in plants and the properties that enable the distribution and abundance of vascular plants in Antarctica. Considering the strong climate changes experienced in the Antarctic Peninsula that include rapid warming during the last decades, and a pause of that warming during recent years (Turner *et al.*, 2016), the next challenge should be to assess the effect of different durations of long-term exposure to warmer temperatures on the photosynthetic performance of *D. antarctica* and *C. quitensis*.

## Supplementary data

Supplementary data are available at *JXB* online.

Table S1. ANOVA of the effects of populations, measurement temperature, and their interaction.

Fig. S1. *Deschampsia antarctica* (left) and *Colobanthus quitensis* (right).

Fig. S2. The response of the net photosynthetic CO_2_ assimilation rate (*A*_N_) to varying internal CO_2_ concentration (*C*_i_).

Fig. S3. Transverse section of the mesophyll and mesophyll cells of *D. antarctica*.

Fig. S4. Transverse section of the mesophyll and mesophyll cells of *C. quitensis*.

Fig. S5. The response of the net photosynthetic CO_2_ assimilation rate (*A*_N_) to varying chloroplast CO_2_ concentration (*C*_c_).

Fig. S6. The relationship between electron transport rate and gross photosynthesis ratio (ETR/*A*_G_) and CO_2_ concentration at the site of carboxylation (*C*_c_).

## Supplementary Material

supplementary_figures_S1_S6_Table_S1
