# Supplementary material for: Photosynthetic limitations in two Antarctic vascular plants: importance of leaf anatomical traits and Rubisco kinetic parameters
Source: J Exp Bot. 2017 May 10;68(11):2871–83. doi: 10.1093/jxb/erx148 (PMC5854023; doi:10.1093/jxb/erx148)
Supplement: supplementary_figures_S1_S6_Table_S1 [file erx148_suppl_supplementary_figures_s1_s6_table_s1.pdf]

Title: Photosynthetic limitations in two Antarctic vascular plants: Importance of leaf anatomical traits and Rubisco kinetics parameters

Authors: Patricia L. Sáez[1*], León A. Bravo[2], Lohengrin A. Cavieres[3], Valentina Vallejos[1], Carolina Sanhueza[3], Marcel Font-Carrascosa[4], Eustaquio Gil-Pelegrín[5], José Javier Peguero-Pina[5], Jeroni Galmés[4]

Supplementary figure legends

Figure S1. *Deschampsia antarctica* (left) and *Colobanthus quitensis* (right) growing *in situ* at King George Island.

Figure S2. The response of net photosynthetic CO_2 assimilation rate (A_N) to varying internal CO_2 concentration (C_i) of *D. antarctica* (A and C) and *C. quitensis* (B and D) in King George (KGI) and Lagotellerie Island (LAG), measured at 10 °C (white circles) and 15 °C (grey circles). Values are means ± S.E. (*n* = 5-7).

Figure S3. Transverse section of the mesophyll (adaxial, Ad and abaxial, Ab surface) and mesophyll cells of *D. antarctica* growing in King George (left) and Lagotellerie Island (right). Chloroplast (Chl) with nearby mitochondria (M), intercellular air space (Ic) and cell wall (Cw).

Figure S4. Transverse section of the mesophyll (adaxial, Ad and abaxial, Ab surface) and mesophyll cells of *C. quitensis* growing in King George (left) and Lagotellerie Island (right). Chloroplast (Chl) with nearby mitochondria (M), intercellular air space (Ic) and cell wall (Cw).

Figure S5. The response of net photosynthetic CO_2 assimilation rate (A_N) to varying chloroplast CO_2 concentration (C_c) of *D. antarctica* (A and C) and *C. quitensis* (B and D) in King George (KGI) and Lagotellerie Island (LAG), measured at 10 °C (white circles) and 15 °C (grey circles). Values are means ± S.E. (*n* = 5-7).

Figure S6. The relationship between electron transport rate and gross photosynthesis ratio (ETR/A_G) and CO_2 concentration at the site of carboxylation (C_c) in *D. antarctica* (white circles) and *C. quitensis* (black circles) growing in King George (KGI) and Lagotellerie Island (LAG), measured at 10 °C or 15 °C. Regression coefficients and significance of the relationship are shown. Values are means ± S.E. (*n* = 5-7).

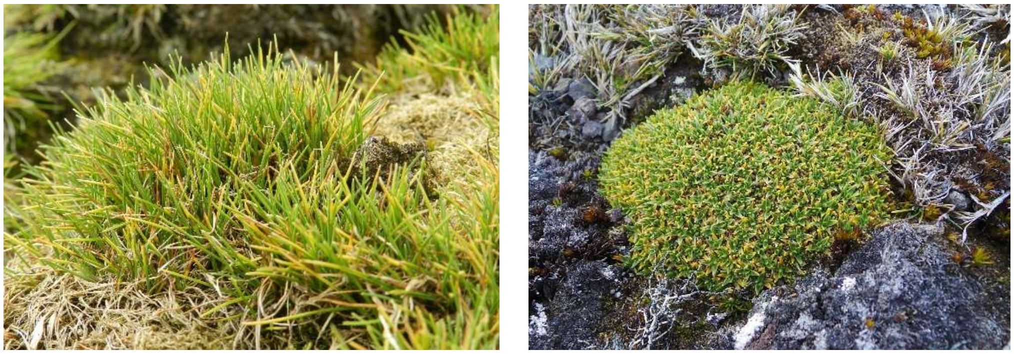

Figure S1.

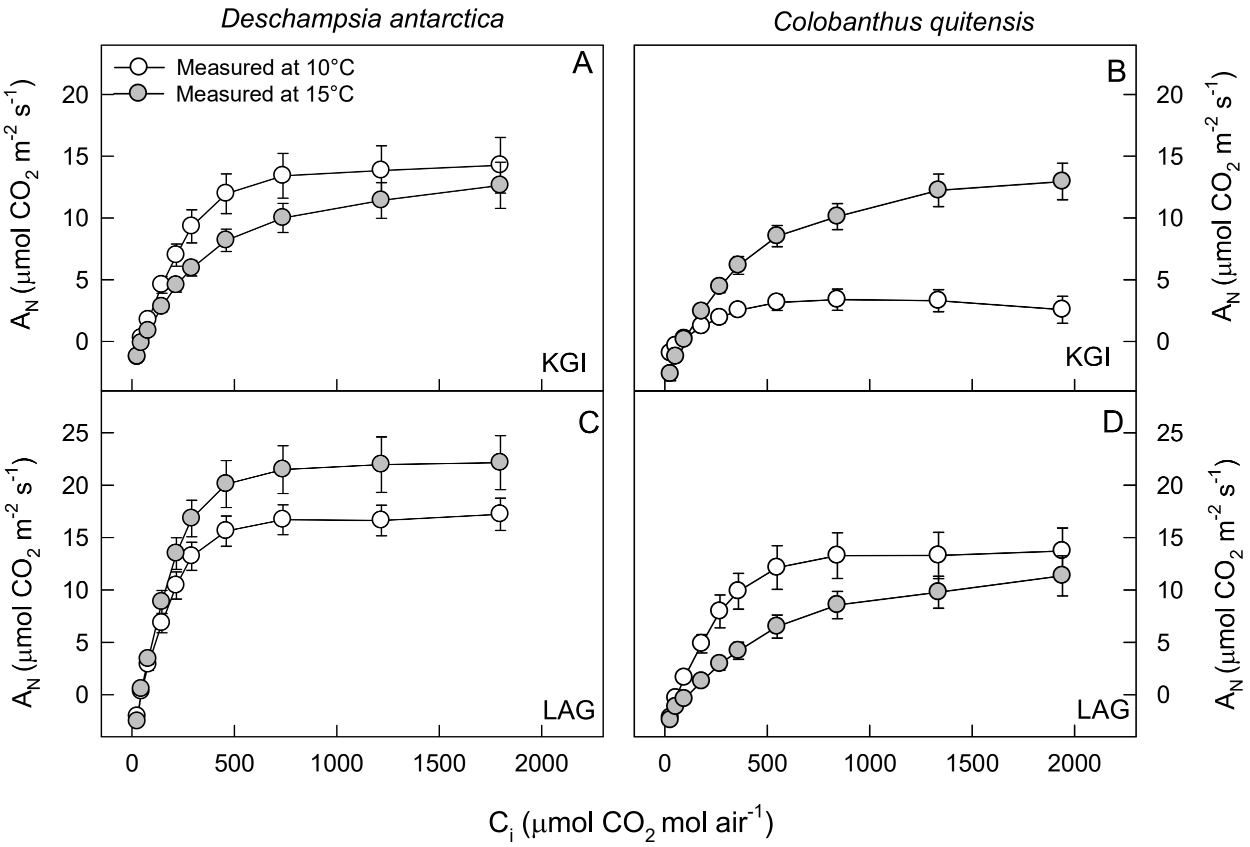

Deschampsia antarctica
Colobanthus quitensis
Measured at 10°C
Measured at 15°C
A
B
C
D
KGI
KGI
LAG
LAG
A_N (µmol CO_2 m^{-2} s^{-1})
C_i (µmol CO_2 mol air^{-1})

Figure S2.

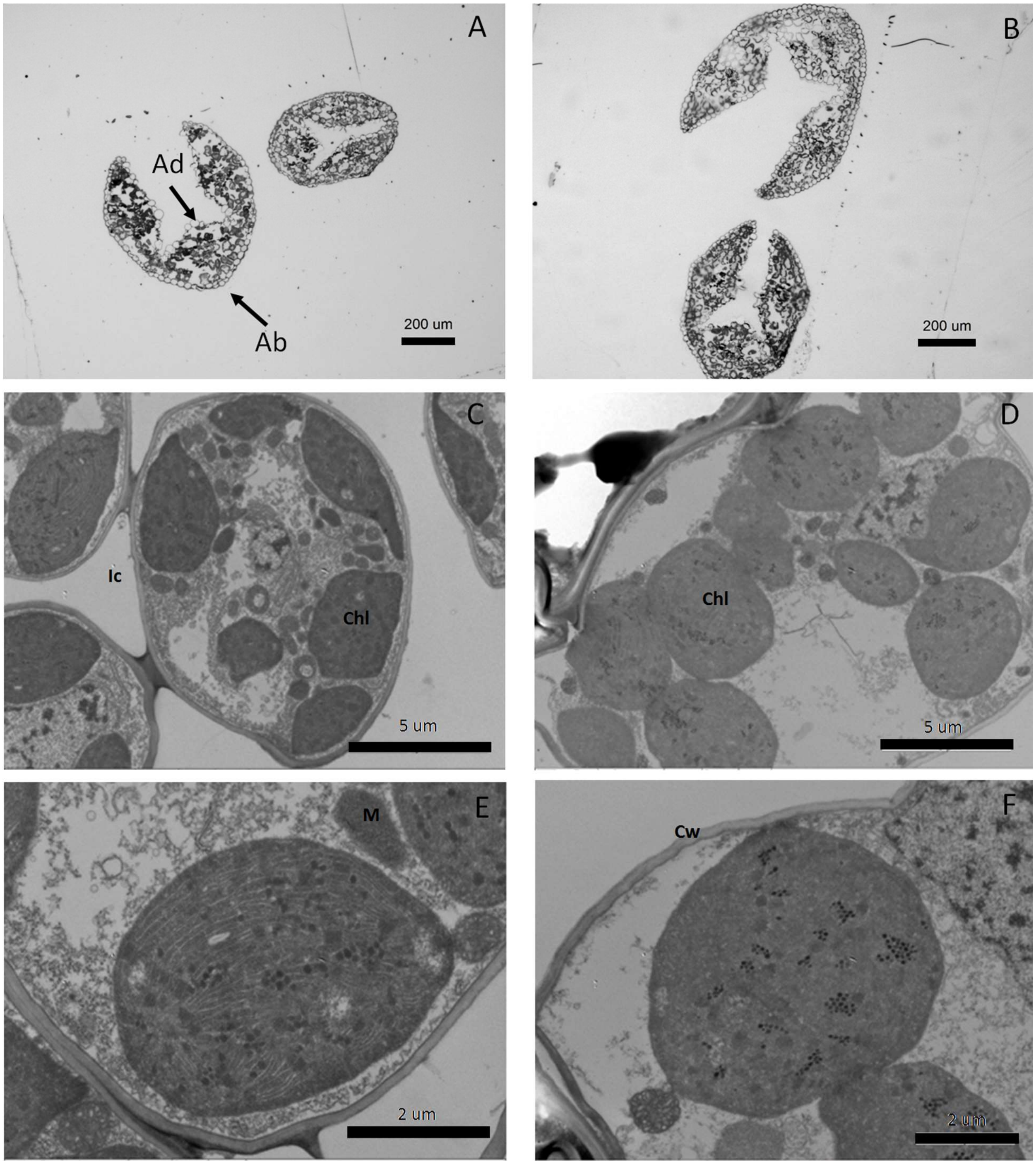

Figure S3.

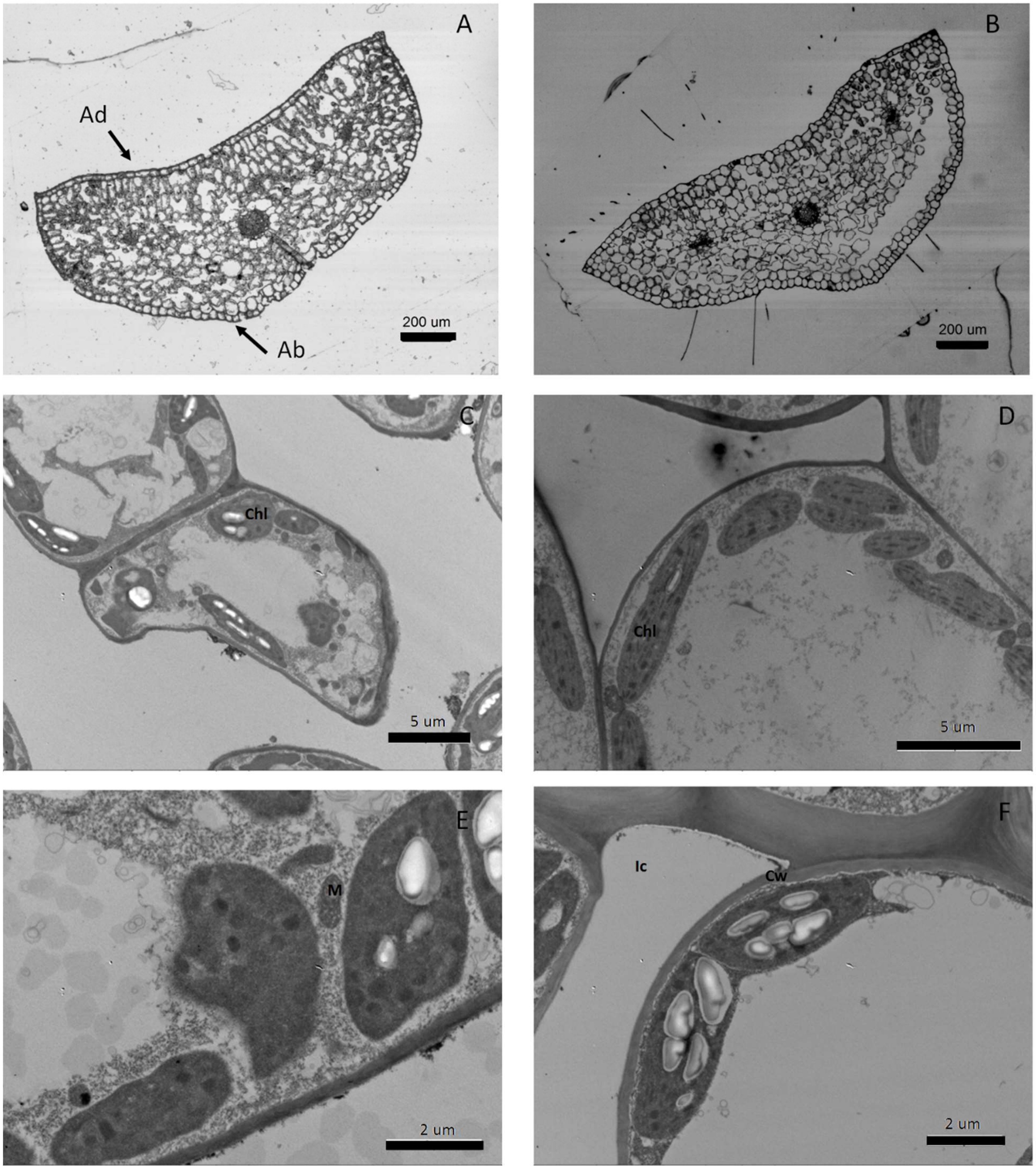

Figure S4.

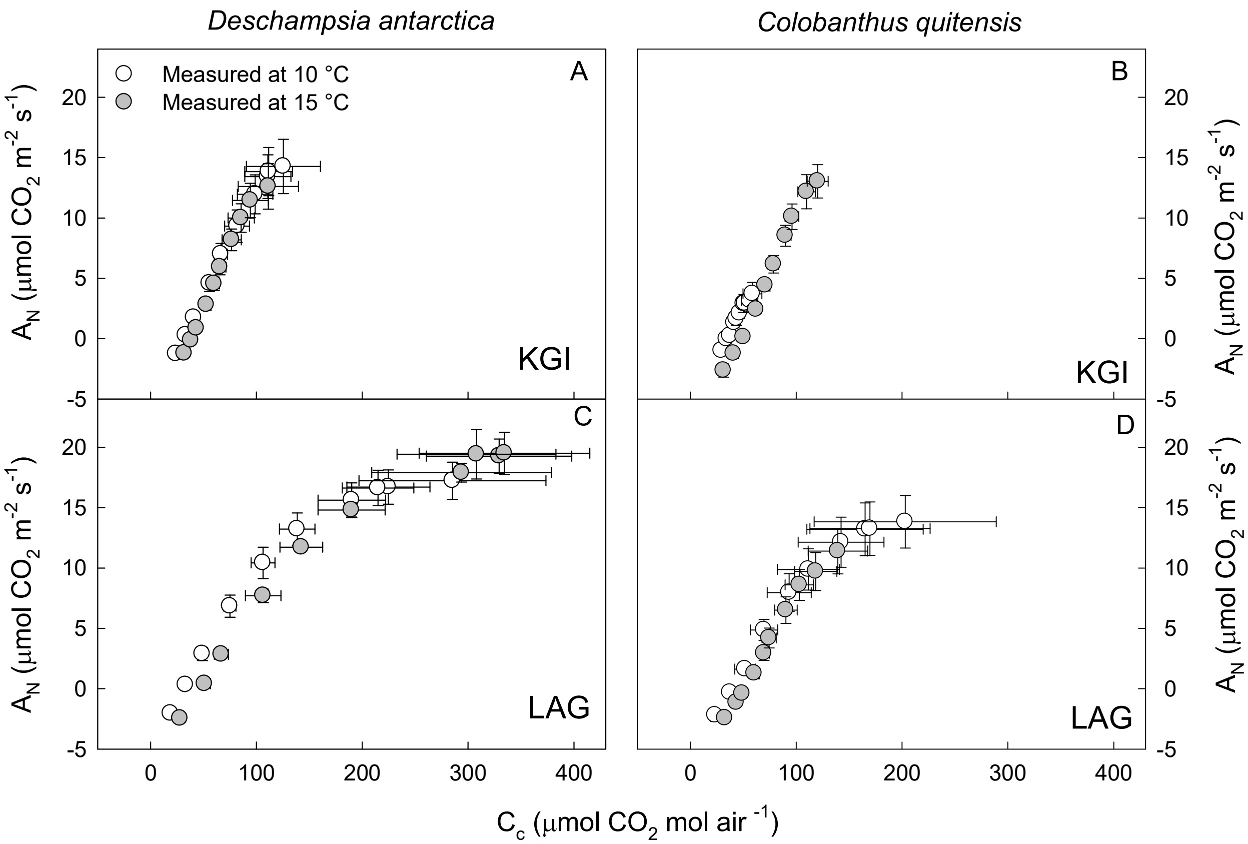
Deschampsia antarctica
Colobanthus quitensis
Measured at 10 °C
Measured at 15 °C
A
B
C
D
KGI
KGI
LAG
LAG
A_N (µmol CO_2 m^-2 s^-1)
C_c (µmol CO_2 mol air ^-1)
-5
0
5
10
15
20
0
100
200
300
400

Figure S5.

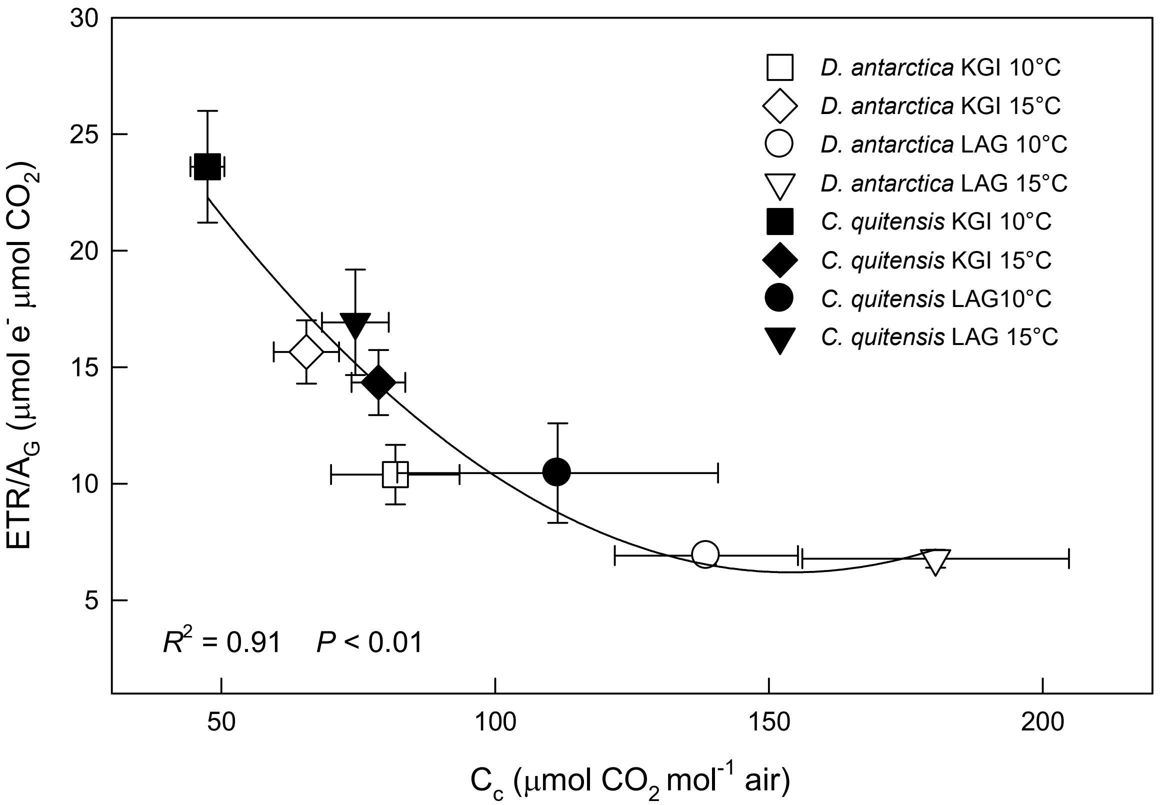

30
25
20
15
10
5
50
100
150
200
ETR/A_G (μmol e⁻ μmol CO_2)
C_c (μmol CO_2 mol⁻¹ air)
D. antarctica KGI 10°C
D. antarctica KGI 15°C
D. antarctica LAG 10°C
D. antarctica LAG 15°C
C. quitensis KGI 10°C
C. quitensis KGI 15°C
C. quitensis LAG10°C
C. quitensis LAG 15°C
R2 = 0.91 P < 0.01

Figure S6.

Table S1. ANOVA of the effects populations (P), temperature of measurement (T) and their interaction on the net photosynthetic CO_2 assimilation rate (A_N), dark respiration (R_{dark}), stomatal (g_s) and leaf mesophyll (g_m) conductances to CO_2, maximum rate of Rubisco carboxylation (V_{cmax}) and the ratio of the electron transport rate and the gross photosynthesis (ETR/A_G) of *D. antarctica* and *C. quitensis* from King George and Lagotellerie Island measured at 10 °C or 15 °C. ns: non-significant.

Parameter	P		T		P × T	
	$F_{(1,19)}$	*P*-value	$F_{(1,19)}$	*P*-value	$F_{(1,19)}$	*P*-value
Deschampsia antarctica						
A_N	35.2	<0.0001	0.46	ns	5.7	<0.05
R_{dark}	0.05	ns	4.49	ns	0.15	ns
g_s	33.9	<0.001	0.04	ns	4.2	ns
g_m	21.7	<0.01	0.8	ns	4.4	<0.05
V_{cmax}	0.8	ns	12.8	<0.001	0.6	ns
ETR/A_G	29.4	<0.001	5.1	<0.05	5.6	<0.05
Colobanthus quitensis						
A_N	8.3	<0.05	1.2	ns	24.8	<0.001
R_{dark}	0.08	ns	6.4	<0.05	0.001	ns
g_s	0.01	ns	0	ns	6	<0.05
g_m	11.9	<0.01	2.9	ns	19.9	<0.001
V_{cmax}	1.3	ns	1.5	ns	17.9	<0.01
ETR/A_G	5.9	<0.05	0.4	ns	13	<0.01
